# Effect of otago exercise on falls in patients with osteoarthritis: A protocol for systematic review and meta-analysis

**DOI:** 10.1097/MD.0000000000023559

**Published:** 2020-12-11

**Authors:** Chao Xie, Weini Wang, Jingfang Pei, Haiyan Wang, Honglin Lv

**Affiliations:** a Department of Spinal Surgery, Yantai Yuhuangding Hospital; b Department of Pediatrics, Women and children's branch Hospital of Muping District Hospital of Traditional Chinese Medicine; c Department of Medical Examination Center; d Central Laboratory, Yantai Yuhuangding Hospital, Yantai, Shandong, China.

**Keywords:** falls, meta-analysis, osteoarthritis, otago exercise, protocol, systematic review

## Abstract

**Background:**

Osteoarthritis (OA) is considered as an established risk factor for falls, while exercise can effectively prevent falls. However, whether otago exercise can prevent falls in OA patients is still controversial. Based on sufficient clinical studies, this study aimed to apply meta-analysis to evaluate the effectiveness of otago exercise on preventing falls in OA patients with.

**Methods:**

PubMed, EMbase, Web of Science and Cochrane Library were searched to collect randomized controlled trial (RCT) of the effect of Otago exercise on falls in OA patients. The search time limit was from the establishment of the database to September 2020. After the 2 researchers independently screened the literature, the data was extracted and the bias risk included in the study was evaluated. Meta-analysis was carried out with RevMan 5.3software.

**Results:**

The results of our meta-analysis could be published in peer-reviewed journals.

**Conclusion:**

This study provided high-quality evidence to support the effect of Otago exercise on falls in OA patients.

**OSF Registration number:**

DOI 10.17605/OSF.IO/Z5XGV.

## 1 Introduction

A fall refers to a sudden, involuntary, unintentional change in posture on the ground or a lower plane.^[1]^ Fall is an important event in clinical nursing and it is caused by decreased balance that is one of the important causes of disability and death in the elderly. About 10% of falls cause serious complications, such as fractures and craniocerebral injuries.^[2]^

As a common and frequently-occurring disease in clinic, osteoarthritis (OA) is an important cause of chronic motor dysfunction and pain in the elderly.^[3,4]^ Theoretically, OA may lead to the decrease of proprioceptive function and sensory input, thus affecting patients ability to maintain balance.^[5,6]^ Therefore, how to prevent patients from falling is the focus of health care workers.

Exercise training can effectively reduce the incidence of falls and improve the balance function of patients. Otago exercise was formulated by CAMPBELL and Otago Medical College of New Zealand in 1997.^[7]^ It aims to prevent the fall of the elderly by guiding the elderly to carry out individual and step-by-step muscle strength and balance function exercise at home. At present, the sport has been applied in many countries and regions, achieving good therapeutic results. Clinical studies proved that Otago exercise can effectively reduce the incidence of falls in the elderly, and improve its balance function and fall efficiency.^[8–10]^

However, the preventive effect of Otago exercise on falls in OA patients is still controversial. In this study, in order to provide evidence-based evidence for health education and reliable reference for community nursing practice, meta-analysis was adopted to evaluate the effect of Otago exercise on fall prevention in OA patients.

## 2 Methods

### 2.1 Study registration

This systematic review protocol was registered in the OSF (DOI 10.17605/OSF.IO/Z5XGV). The protocol refers to the guide book of Preferred Reporting Items for Systematic Reviews and Meta-Analyses Protocols (PRISMA-P).^[11]^

### 2.2 Inclusion criteria for study selection

#### 2.2.1 Type of studies

All RCT about the impact of Otago exercise on OA falls was included. Retrospective studies, case reports, observational studies, animal studies, repeatedly published studies, and studies that do not have complete data were be excluded.

#### 2.2.2 Types of participants

All participants who met the diagnostic criteria for osteoarthritis were included, regardless of their age, sex, and race

#### 2.2.3 Types of outcome measures

Main outcome: fall rate.

Secondary outcome:

1. Single-leg standing test.
2. Berg balance scale.
3. Timed up and go.
4. Chair standing test.

### 2.3 Search methods for identification of studies

PubMed, EMbase, Web of Science, and Cochrane Library were search to collect RCT of the effect of Otago exercise on falls in OA patients. The search time limit is from the establishment of the database to September 2020. Meanwhile, the clinical trial registration websites (http://www.ClinicalTrial.gov and http://www.chictr.org.cn) were searched. The references included in the literature were searched manually to supplement and obtain relevant literatures. The retrieval was carried out by the way of subject words combined with free words. Taking PubMed as an example, the specific retrieval strategy is displayed in Table 1.

**Table 1:** Search strategy in PubMed database.

Number	Search terms
#1	Osteoarthritis[MeSH]
#2	Osteoarthritides[Title/Abstract]
#3	Osteoarthrosis[Title/Abstract]
#4	Osteoarthroses[Title/Abstract]
#5	Arthritis, Degenerative[Title/Abstract]
#6	Arthritides, Degenerative[Title/Abstract]
#7	Degenerative Arthritides[Title/Abstract]
#8	Degenerative Arthritis[Title/Abstract]
#9	Arthrosis[Title/Abstract]
#10	Arthroses[Title/Abstract]
#11	Osteoarthrosis Deformans[Title/Abstract]
#12	or/1–11
#13	Otago[Title/Abstract]
#14	Accidental Falls[MeSH]
#15	Falls[Title/Abstract]
#16	Falls, Accidental[Title/Abstract]
#17	Accidental Fall[Title/Abstract]
#18	Fall, Accidental[Title/Abstract]
#19	or/14–18
#20	#12 and #15 and #19

### 2.4 Data extraction, quality, and validation

#### 2.4.1 Study selection and inclusion

**Figure 1:**
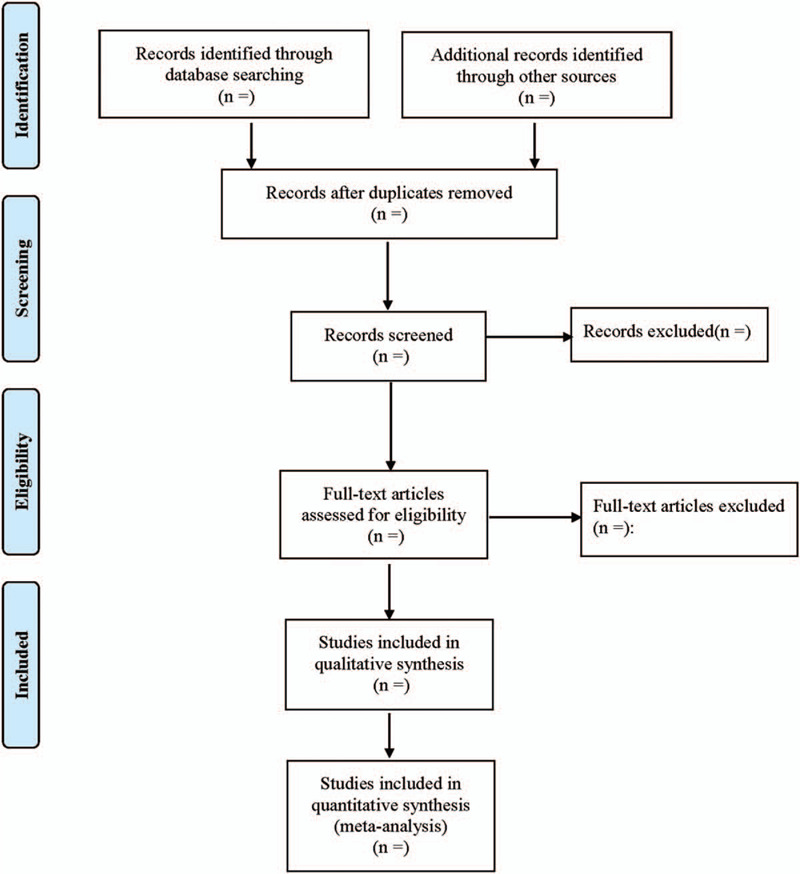
Flow diagram of study selection process.

When screening the literature, first reading the title. After excluding the obviously irrelevant literatures, further reading the abstract and the full text to determine whether to include or not. If necessary, contacting the original research author by email or telephone to obtain undetermined information that is very important to this study. Any differences would be resolved by consensus. Finally, another researcher would resolve the inconsistency and examine the final inclusion of literatures. The research selection process is illustrated in Figure 1

#### 2.4.2 Data extraction and management

The contents of data extraction include:

1. In the study, the basic information include the first author, study area and publication time.
2. The baseline characteristics of the subjects include samples and age.
3. Details of intervention measures.
4. Key elements of bias risk assessment.
5. Concerned outcome indicators and measurement data.

It would be screened independently by 2 researchers, and if there are any differences, they would be resolved through discussion or negotiation with a third party.

#### 2.4.3 Assessment of risk of bias

Two independent evaluators applied RevMan 5.3, a tool recommended by Cochrane collaboration Network, to evaluate the bias risk of included literatures. The assessments include:

1. Whether the generation and distribution of random sequences are hidden?
2. Whether participants and interventionists implement blind methods?
3. Whether blind methods are applied to evaluators?
4. Whether the resulting data are completed?
5. Whether the research results are selectively reported?
6. Other biases.

The score was divided into 3 grades, expressed as low-risk, unclear, and high-risk. If 2 evaluators have disputes in the process of quality evaluation, the third evaluator is invited to participate in the discussion, so as to form a unified opinion.

### 2.5 Quantitative data and statistical methods

#### 2.5.1 Quantitative data synthesis

Meta-analysis was carried out with RevMan 5.3 software. The data included in the study was continuous, standardized mean difference (SMD), and 95% confidence interval (CI) analysis. The two-category variable applied risk ratio (RR) as the effect analysis statistic, and each effect quantity provides its 95% CI.

If there is no statistical heterogeneity among the results (*I*^2^ < 50%, *P* > .1), the fixed effect model is adopted for meta-analysis. If *P* < .1, and *I*^2^ ≥ 50%, there is a large heterogeneity among the results, and the causes of heterogeneity are analyzed. If there is obvious clinical heterogeneity, subgroup analysis, or sensitivity analysis can be carried out based on its source. In the absence of significant clinical and methodological heterogeneity, statistical heterogeneity would be considered and analyzed with a random effect model.

#### 2.5.2 Assessment of heterogeneity

The heterogeneity among the included results was analyzed by χ^2^ test (the test level was α = 0.1). At the same time, the heterogeneity was quantitatively judged by *I*^2^.

#### 2.5.3 Assessment of reporting bias

If no less than 10 studies are included,^[12,13]^ we would use funnel chart method to detect publication bias.

#### 2.5.4 Subgroup analysis and investigation of heterogeneity

We conducted a subgroup analysis to evaluate factors that may lead to clinical heterogeneity, including age, region, sample size, duration of intervention, follow-up time, and so on.

#### 2.5.5 Sensitivity analysis

After eliminating each of the included studies one by one, the effects were combined to evaluate the robustness and reliability of the results of meta-analysis.

#### 2.5.6 Grading the quality of evidence

Grading of Recommendation Assessment, Development and Evaluation system recommendation classification method was adopted to evaluate the evidence grade. The quality of evidence can be divided into 4 grades: high quality, medium quality, low quality, and very low quality. Although evidence based on RCTs is defaulted to high quality, 5 downgrade factors are still needed to be considered, namely, the limitations of the study, inconsistencies in the results of the study, indirect evidence, inaccuracy, and publication bias.

### 2.6 Dealing with missing data

Missing data was excluded. Only analyzing the existing data and discuss the impact on the whole.

### 2.7 Ethics and dissemination

The meta-analysis of this article does not involve moral cognition or ethical review and will be presented in the form of printed matter or related meetings.

## 3 Discussion

Current studies revealed that 75% of physical falls occurs in the elderly due to pain and muscle weakness by degenerative OA of knees and ankles.^[14]^ Falls and OA usually coexist in the elderly.^[15]^ OA is usually associated with dyskinesia for pain and muscle weakness and is therefore considered to be an established risk factor for falls.

Exercise benefits OA patients by reducing pain and improving mobility.^[16,17]^ Therefore, with any fall prevention program, providing an acceptable level of appropriate exercise is critical to ensure its success. In addition, interventions can simultaneously reduce multiple existing risk factors for falls, such as fear of falls and gait and balance problems, which should be encouraged.

Otago exercise consists of 2 parts of training. The first part includes flexibility training, lower limb muscle strength training, and balance function training. The second part is walking training. In the training manual, there is a standard instruction for each training action, and the requirements for the time, intensity and frequency of the exercise are clear. The main purpose of Otago exercise is to improve the static and dynamic balance of OA patients through targeted limb strength training and balance function training, so as to enhance the confidence of patients to perform activities in complex environment. This study reports the accordance with the requirements of the PRISMA statement, and provides evidence of higher methodological quality and more rigorous reporting quality for the effects of Otago exercise on falls and balance in patients suffering from OA.

## Author contributions

**Data curation:** Weini Wang.

**Formal analysis:** Jingfang Pei.

**Funding acquisition:** Honglin Lv.

**Methodology:** Haiyan Wang.

**Project administration:** Honglin Lv.

**Software:** Weini Wang.

**Supervision:** Chao Xie.

**Validation:** Honglin Lv.

**Visualization:** Weini Wang.

**Writing – original draft:** Honglin Lv, Chao Xie.

**Writing – review & editing:** Honglin Lv, Chao Xie.
